# Nanoflake NiMn Layered Double Hydroxide Coated on Porous Membrane-like Ni-Foam for Sustainable and Efficient Electrocatalytic Oxygen Evolution

**DOI:** 10.3390/membranes13090748

**Published:** 2023-08-22

**Authors:** Verjesh Kumar Magotra, Arjun Magotra, Sawanta S. Mali, Hee C. Jeon, Tae W. Kang, Amol S. Salunke, Chang Kook Hong, Nabeen K. Shrestha, Hyunsik Im, Akbar I. Inamdar

**Affiliations:** 1 Quantum-Functional Semiconductor Research Center, Dongguk University, Seoul 13557, Republic of Korea; 2 Department of Computer Science and Engineering, Faculty of Engineering and Technology, Jain (Deemed-to-be University), Bengaluru 562112, India; 3 Polymer Energy Materials Laboratory, School of Applied Chemical Engineering, Chonnam National University, Gwangju 500757, Republic of Korea; 4 Division of Physics and Semiconductor Science, Dongguk University, Seoul 04620, Republic of Korea

**Keywords:** electrocatalysis, water splitting, NiMn LDHs, heterostructure catalysis, oxygen evolution reaction

## Abstract

Layered double hydroxides (LDHs) have gained vast importance as an electrocatalyst for water electrolysis to produce carbon-neutral and clean hydrogen energy. In this work, we demonstrated the fabrication of nano-flake-like NiMn LDH thin film electrodes onto porous membrane-like Ni-foam by using a simple and cost-effective electrodeposition method for oxygen evolution reaction (OER). Various Ni_1-x_Mn_x_ LDH (where x = 0.15, 0.25, 0.35, 0.50 and 0.75) thin film electrodes are utilized to achieve the optimal catalyst for an efficient and sustainable OER process. The various composition-dependent surface morphologies and porous-membrane-like structure provided the high electrochemical surface area along with abundant active sites facilitating the OER. The optimized catalyst referred to as Ni_0.65_Mn_0.35_ showed excellent OER properties with an ultralow overpotential of 253 mV at a current density of 50 mAcm^−2^, which outperforms other state-of-the art catalysts reported in the literature. The relatively low Tafel slope of 130 mV dec^−1^ indicates faster and more favorable reaction kinetics for OER. Moreover, Ni_0.65_Mn_0.35_ exhibits excellent durability over continuous operation of 20 h, indicating the great sustainability of the catalyst in an alkaline medium. This study provides knowledge for the fabrication and optimization of the OER catalyst electrode for water electrolysis.

## 1. Introduction

The global dependence on non-renewable energy sources like oil, gas and coal has created serious environmental issues like air and water pollution. Therefore, meeting the increased demand for clean, sustainable, and environmentally friendly energy sources forces researchers to develop alternatives to fossil fuels. Hydrogen is one of the alternatives, which possesses outstanding properties such as high efficiency, sustainability, nontoxicity, a high gravimetric energy density, and carbon neutrality to the environment [1,2,3]. Electrocatalysis is an emerging and advanced technology over steam reforming of natural gas, the oxidation of methane, and biomass and coal gasification because of its inexpensiveness and cleanest production [4,5,6,7,8]. In this process, a strong bond of water is broken to form molecular oxygen and hydrogen with the help of catalysts. It consists of two reactions, namely hydrogen evolution reaction (HER at the cathode) and oxygen evolution reaction (OER at the anode) [9,10,11]. To break water into oxygen and hydrogen, a theoretical voltage required is 1.23 V. However, water electrolysis requires added voltage, which is called overpotential. This overpotential is due to the slower OER kinetics at the electrode–electrolyte interface [12]. Valuable metals like RuO_2_ and IrO_2_ are efficient and active, but they are very expensive in the market, which limits their use for large production [13,14]. It is thus essential to fabricate cheap, efficient and sustainable electrocatalysts that are abundant in natural elements, readily available on a large scale and can operate at low overpotentials.

Over the last two decades, various catalysts associated with mixed, complex, bimetallic alloys, hydroxides, sulfides and nitrides have been investigated as electrocatalysts for water splitting [15,16,17,18,19,20,21,22,23,24,25,26,27,28]. In these studies, various strategies like morphological engineering, composition tuning between constituent elements, alloy formation, and heterostructure formation have been employed to enhance the catalytic sites. Recently, the fabrication of mixed bimetallic alloys has attracted particular interest due to its advantages like tuning of the local electronic structure by varying the composition of the elements, generation of the electrochemically active catalytic sites and morphological modification to enhance the adsorption of the water molecules [16,29,30,31]. In this category, a bimetallic layered double hydroxide (LDH) attracted much attention due to its outstanding properties including being readily available, having a large physical surface area, ease to tune the composition, and having a stacked structure. Moreover, the catalytic performance can be enhanced by increasing active sites by intentionally introducing a defect in the LDHs. From the literature survey, it is noted that the LDHs based on NiFe, CoFe, and CuFe have been investigated widely and proved their ability for efficient water-splitting activity and remarkable durability [2,25,32]. Though a huge number of metal oxide/hydroxide catalysts have been studied until now, they suffer from poor stability and high overpotentials. It forces researchers to investigate other bimetallic electrocatalysts like NiMn-based LDHs, which are sparsely reported in the literature. A few studies suggest that NiMn-based LDHs can be easily synthesized and may provide enhanced catalytic performance [33,34,35,36]. Moreover, several kinds of membranes have also played a very important role in water-splitting electrolysis technologies [1]. Different nanostructures of the NiMn LDHs such as ultrathin nanosheets and peony-flower-like heterostructures are reported as efficient catalysts for OER [37,38]. Moreover, the atomic structure of several XY-LDHs (where X = Ni, Co and Y = Co, Fe, Mn) is controlled by controlling the synthesis strategies, and its intrinsic activity is investigated [39]. In this, Mn is used as a dopant element to tune the electronic structure of the host catalysts, in which the Mn^3+^ state is proven to be an active site for water oxidation and to have a porous-membrane-like structure, allowing faster accesses to expose electrolytes. Thus, from the above discussion, it has been concluded that NiMn LDH will be the potential OER candidate for water electrolysis.

In this study, we demonstrated the fabrication of the bimetallic Ni_1−x_Mn_x_ LDH (where x = 0.15, 0.25, 0.35, 0.50 and 0.75) thin film electrodes via a simple electrodeposition method on membrane-like Ni foam as an efficient catalyst for OER. The composition between Ni and Mn was systematically varied to achieve different morphologies and electronic structure modifications in the catalyst. The synergetic effect of the structural and morphological modification on the oxygen evolution reaction is examined in an alkaline medium with 1 M KOH electrolyte. The best-performing Ni_0.65_Mn_0.35_ showed the lowest overpotential of 253 mV at 50 mAcm^−2^. The Tafel analysis revealed that Ni_0.65_Mn_0.35_ has promising reaction kinetics for OER. The catalyst is highly stable in an alkaline medium for long-term operation, suggesting its applicability for mass production of hydrogen via water electrolysis.

## 2. Experimental Section

### 2.1. Fabrication of NiMn LDH Thin-Film Electrode

Nanoflake-like Ni_1−x_Mn_x_ LDH (where x = 0.15, 0.25, 0.35, 0.50 and 0.75) catalyst electrodes were fabricated on a membrane-like Ni foam using an electrodeposition method. It consists of a working electrode, counter electrode and reference electrode, which are Ni foam, Pt wire and SCE, respectively. Precursor chemicals made by Sigma Aldrich (St. Louis, MO, USA) like Ni (II) chloride hexahydrate and Mn (II) chloride were used without further purification. Before the deposition, the Ni foam substrates were properly cleaned with an acid solution and dried in the oven for 24 h. A solution of 100 mL was prepared using nickel chloride and manganese chloride, and it was stirred for 5 min to ensure it was mixed properly. A chronoamperometric technique was used to fabricate the thin film electrodes at a constant applied voltage of 0.5 V (vs SCE) for 1.0 h. Figure S1 (Supplementary Information) shows the typical chronoamperometric curves for the electrodeposition of Ni_1−x_Mn_x_ LDH thin film electrodes. Various electrodes with different molar ratios of Ni and Mn such as 0.085:0.015, 0.075:0.025, 0.065:0.035, 0.500:0.050 and 0.025:0.075 were fabricated, and they were denoted as Ni_0.85_Mn_0.15,_ Ni_0.75_Mn_0.25,_ Ni_0.65_Mn_0.35_, Ni_0.50_Mn_0.50_ and Ni_0.25_Mn_0.75_, respectively. Finally, the prepared electrodes were washed with D. I. water and dried.

### 2.2. Characterization and Electrochemical Measurements of NiMn LDH

The structural properties were studied using X-ray diffraction (XRD) measurements using an X-ray diffractometer (Rigaku, Tokyo, Japan) with the Cu Kα line having a wavelength of 1.5410 Å. Raman spectroscopy was also used to investigate the structural properties of the electrodes using a Raman spectrometer (model name NRS-5100 and serial number B008061420) with an excitation wavelength of 532.13 nm. A field emission scanning electron microscope (FE SEM) (model No. SU-70 and serial number HI-0008-0003) with an accelerating voltage of 15 kV, made by the company Hitachi, Japan, was used to investigate the morphology of the Ni_1−x_Mn_x_ LDH thin film electrodes. The surface chemical oxidation states of the best-performing Ni_0.65_Mn_0.35_ were carried out using X-ray photoelectron spectroscopy (XPS) (VG Multilab 2000, ThermoVG Scientific, made in the UK). For the electrochemical measurements in which NiMn-LDH was a working electrode, the counter electrode was Pt wire, and the reference electrode was a saturated calomel electrode (SCE). A VersaSTAT3 workstation (Princeton Applied Research, Oak Ridge, TN, USA) was used for all electrochemical measurements in an alkaline medium (1 M KOH). The LSV was recorded at a scan rate of 5 mVs^−1^. The electrochemical impedance spectroscopy (EIS) measurement studies were performed between a frequency range of 1 Hz and 10 kHz.

## 3. Results and Discussion

**Figure 1:**
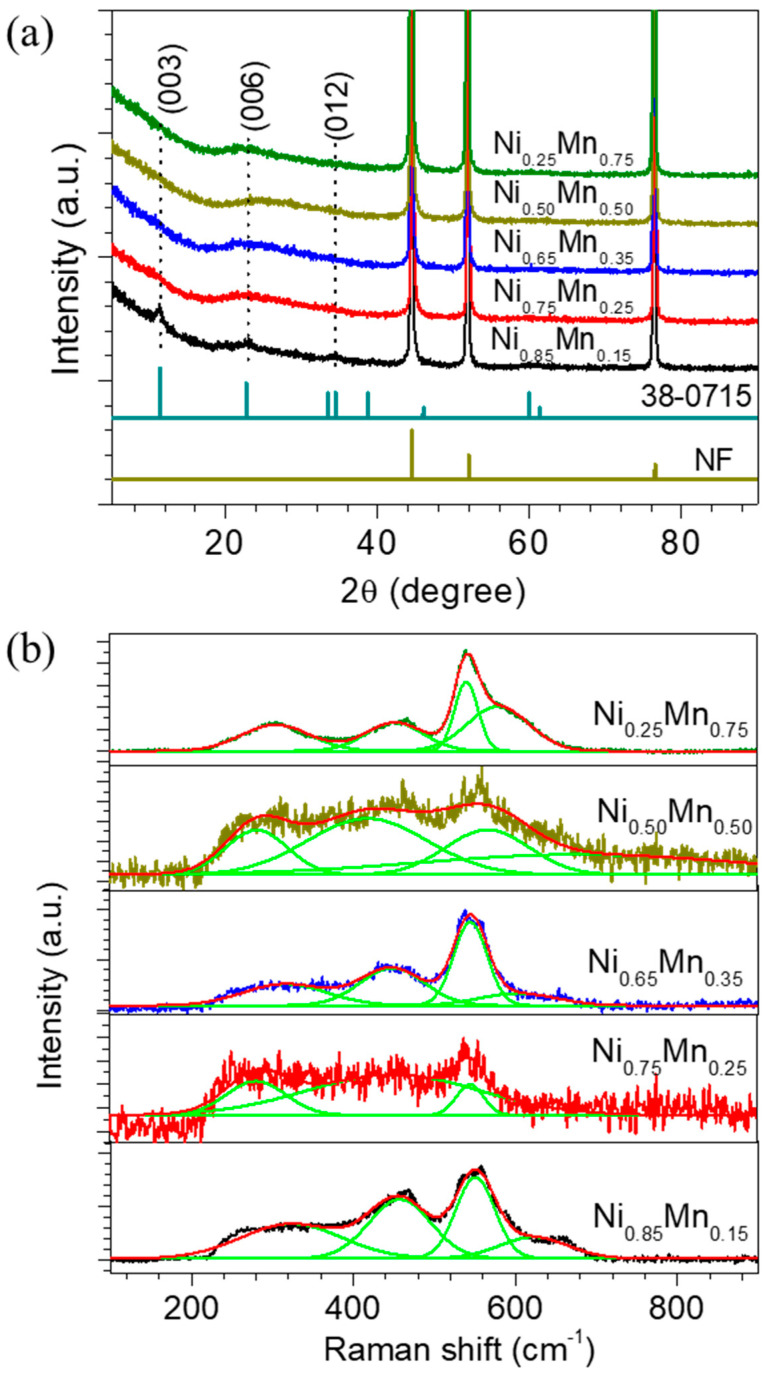
Structural analysis of the Ni_1−x_Mn_x_ LDH thin film electrodes. (**a**) XRD patterns measured in the range of 10° to 80° with a scanning speed of 2° per minute; XRD peaks indicated with star symbol are of Ni−foam substrate. (**b**) Raman spectrum measured in the wavelength range of 100 to 900 cm^−1^.

The structural properties of the electrodeposited Ni_1−x_Mn_x_ LDH thin film electrodes are studied using X-ray diffraction (XRD) and Raman spectroscopy measurements. Figure 1a shows the XRD patterns of the Ni_1−x_Mn_x_ LDHs measured in the 2θ angles between 10° and 80° with a scanning speed of 2° per minute. The major diffraction peaks observed at 44.46°, 51.96° and 76.48° (indicated with a star symbol) with high intensity are related to the Ni foam substrate. The XRD pattern of the sample Ni_0.85_Mn_0.15_ (also shown in Figure S2, Supplementary Information) displayed a clear diffraction peak at 11.20°, 23.04°, and 34.46° (indicated with dotted lines) with miller indices of (003), (006), (012), respectively, which are ascribed to the formation of NiMn LDH (JCPDS No. 38-0715). With an increase in the Mn content, no obvious change occurs in the XRD patterns; moreover, the peak located at 23.04° becomes broad compared to the least Mn content sample (Ni_0.85_Mn_0.15_). This suggests the formation of the nanocrystalline layered structure of the NiMn hydroxide. The XRD results presented in this work are consistent with those reported in the literature without any impurities [36,37,38,39,40,41]. Thus, the above analysis indicates the successful synthesis of the NiMn LDH using the electrodeposition technique.

The Raman analysis provides a crucial understanding of the structural characteristics and surface properties of the NiMn LDH catalysts. Figure 1b shows the Raman spectrum of the Ni_1−x_Mn_x_ LDH thin film electrodes measured in the wavelength range of 100 to 900 cm^−1^. Each spectrum consists of four deconvoluted peaks associated with the specific vibrational modes of the molecule present in the material. The band observed in the range of 400–800 cm^−1^ is typically associated with the vibrational modes of the metal–oxygen bonds in the hydroxide layer. The band located near the wavelength of 453 and 541 cm^−1^ is associated with the vibrations of the Ni-OH and Ni-O stretching modes, respectively [42,43]. The other band, which appeared at 630 cm^−1^, is due to the Mn-O vibrations [44]. Moreover, the peak at nearly 310 cm^−1^ is associated with symmetric and asymmetric stretching of the Mn-O-Mn vibrations [45]. Thus, the Raman analysis indicated that the NiMn LDH exists in composite phases.

**Figure 2:**
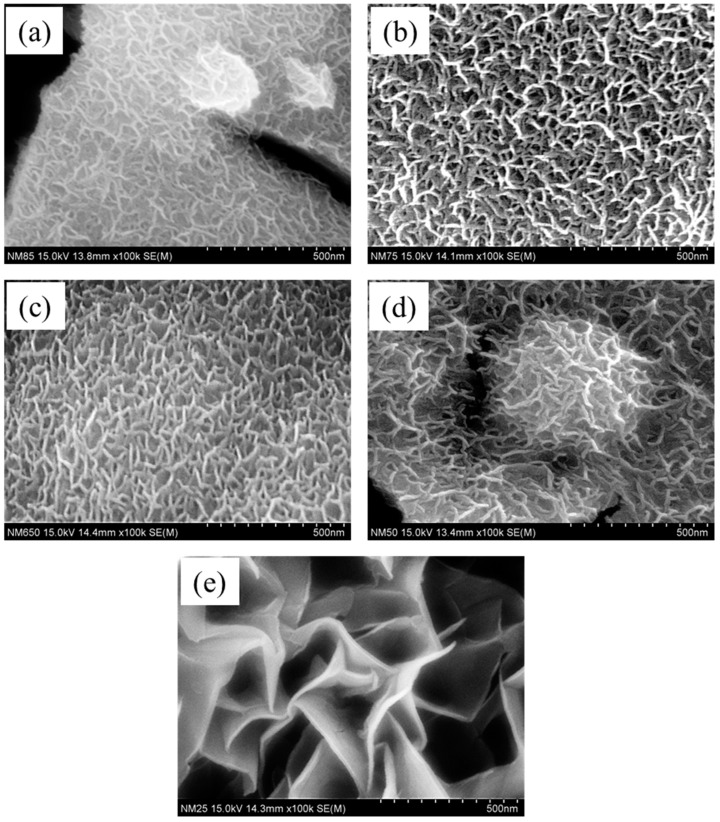
Scanning electron microscopy images of the Ni_1−x_Mn_x_ LDH thin film electrodes. (**a**) Ni_0.85_Mn_0.15,_ (**b**) Ni_0.75_Mn_0.25,_ (**c**) Ni_0.65_Mn_0.35_, (**d**) Ni_0.50_Mn_0.50_ and (**e**) Ni _0.25_Mn _0.75._ All the samples showed nano-flake-like morphologies.

The morphology of the catalyst electrode is an important parameter in the electrocatalysis process, which facilitates adsorption of the water molecules. Therefore, the morphologies of the Ni_1−x_Mn_x_ LDH thin film electrodes are investigated using scanning electron microscopy (SEM) analysis. All of the samples (Figure 2 and Figure S3) exhibited nanostructured nanoflake-like morphologies with diverse lateral sizes providing a high electrochemical surface area, promoting the elevation of water-splitting properties. Ni_0.85_Mn_0.15_ shows (Figure 2a) undergrown interconnected nanoflakes entirely covering the Ni foam substrate. The low magnification view in Figure S3a (Supplementary Information) depicts the existence of many cracks in the membrane-like Ni foam. With an increase in the Mn content, the completely grown nanoflakes are observed in Figure 2b,c for the samples Ni_0.75_Mn_0.25_ and Ni_0.65_Mn_0.35_, respectively. Moreover, the sample Ni_0.50_Mn_0.50_ showed a bed of nanoflakes with nanoflake balls. The Ni_0.25_Mn_0.75_ (Figure 2e) exhibited a card-sheet-like structure with cauliflowers (Figure S3e) on the top. The elemental compositions in the catalyst also have great importance in the modification of the physical and chemical properties of the catalysts. Therefore, the EDX spectra shown for the representative sample Ni_0.85_Mn_0.15_ in Figure S4 (Supplementary Information) indicate the presence of the constituent elements Ni, Mn and O in the sample. Thus, the morphological investigation depicts that samples have nano-natures with the desired elemental composition and may possibly have a high adsorption ability.

**Figure 3:**
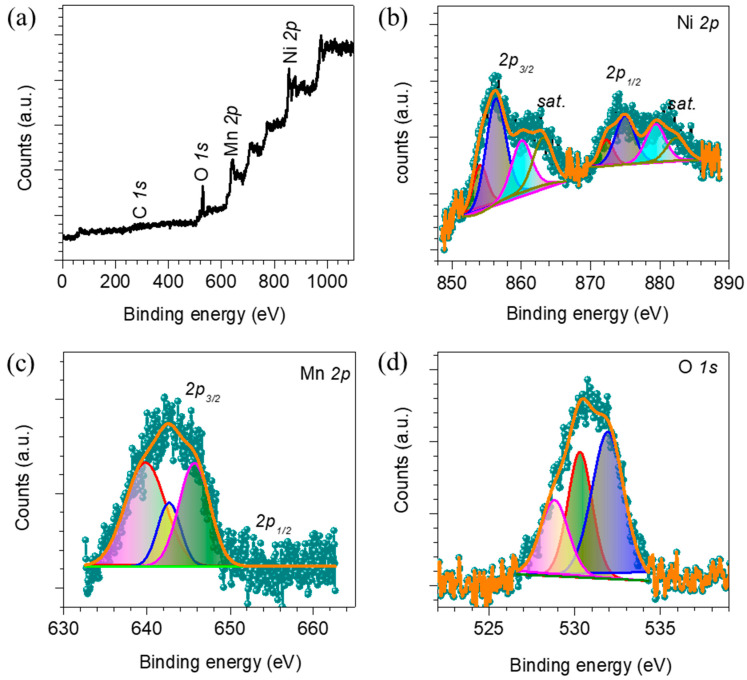
X-ray photoelectron spectroscopy data of the best-performing Ni_0.65_Mn_0.35_, thin film electrode. (**a**) Survey spectra indicating the presence of Ni, Mn, C and O in the catalysts; deconvoluted spectra of the (**b**) Ni 2p, (**c**) Mn 2p, (**d**) O 1s.

Chemical properties such as the surface oxidation states of the best-performing Ni_0.65_Mn_0.35_ thin film catalyst are examined with XPS measurements, which are shown in Figure 3. As predicted, the XPS survey spectrum (Figure 3a) reveals the presence of Ni, Mn, C and O in the sample. The core level Ni 2p XPS spectrum shown in Figure 3b exhibits a peak at binding energies of 854.1, 856.3, 872.5 and 874.8 eV, which are associated with the Ni^2+^ 2p3/2, Ni^3+^ 2p3/2, Ni^2+^ 2p1/2 and Ni^3+^ 2p1/2, respectively, while the other four peaks are satellite peaks [46]. The core level XPS spectrum of the Mn 2p_3/2_ (Figure 3c) shows three deconvoluted peaks at binding energies of 638.9, 642.6 and 645.6 eV, which are ascribed to the existence of the Mn^2+^, Mn^3+^, and Mn^4+^ oxidation states, respectively [47]. Moreover, the deconvoluted O 1s XPS spectrum (Figure 3d) exhibits peaks at binding energies of 528.77, 530.31 and 531.97 eV, which are due to the presence of the metal–oxide, metal–hydroxide (M-OH, where M = Ni, Mn) and adsorbed hydroxyl groups, respectively, [48]. Thus, the XPS analysis confirms the formation of the NiMn LDH in the sample.

**Figure 4:**
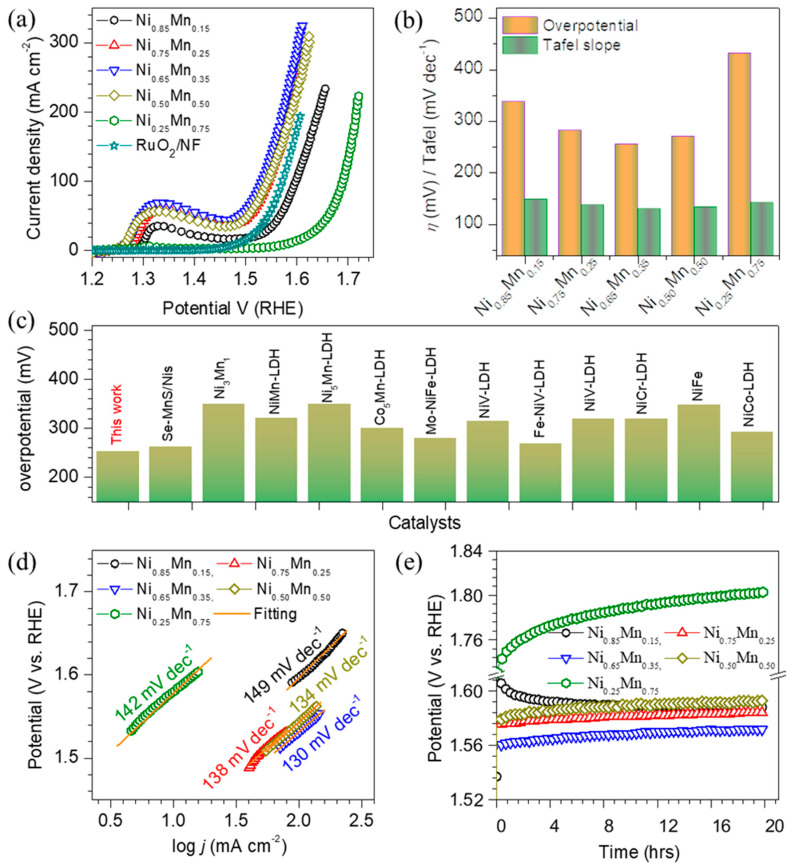
Electrochemical OER properties of the Ni_1−x_Mn_x_ LDH thin film electrodes measured for 1 M KOH electrolyte. (**a**) *iR*−corrected OER polarization curves recorded at a scan rate of 5 mVs^−1^, (**b**) Tafel slopes and overpotentials required to reach a current density of 50 mAcm^−2^, (**c**) comparison of the overpotential observed at 50 mAcm^−2^ with other state−of−the−art catalysts reported in the literature, (**d**) Tafel slopes for the OER, and (**e**) chronopotentiometric stability curves (no iR correction) for the OER properties over 20 h recorded at a current density of 50 mAcm^−2^.

The Ni_1-x_Mn_x_ LDH thin film catalysts are effectively utilized as potential candidates for the oxygen evolution reaction (OER) study. Prior to the actual OER study, all the catalyst electrodes are electrochemically stabilized using cyclic voltammetry techniques for 30 cycles at a scan rate of 100 mVs^−1^ in 1 M KOH electrolyte. After stabilization, all of the catalyst electrodes are studied using LSV measurements for OER. Figure 4a represents the *iR*-corrected polarization curves of NiMn LDH, whereas Figure S5 (Supplementary Information) shows its enlarged view. The systematic shift in the LSV curves with elemental compositions is observed, which requires different overpotentials. The presence of a pair of redox peaks observed near 1.3 V (vs. RHE) in all of the LSV curves is due to the redox transition of Ni^2+^/Ni^3+^. The overpotential is found to be lowest (253 mV) at a current density of 50 mA cm^−2^ for Ni_0.65_Mn_0.35_ catalyst. For other catalysts such as Ni_0.85_Mn_0.15_, Ni_0.75_Mn_0.25_, Ni_0.50_Mn_0.50_ and Ni_0.25_Mn_0.75_, it was found to be 336, 277, 268, and 429 mV, respectively. Figure 4b depicts the graph of overpotentials of the Ni_1−x_Mn_x_ LDH to achieve a current density of 50 mA cm^−2^. The OER parameters obtained at different current densities for the Ni_1−x_Mn_x_ LDH are presented in Table S1 (Supplementary Information). The overpotential observed for Ni_0.65_Mn_0.35_ catalyst is superior to that reported for other catalysts in the literature (Figure 4c) like NiFe (348 mV @ 10mA cm^−2^) [29], Se-MnS/NiS (263 mV @ 50 mA cm^−2^) [33], Ni_3_Mn_1_ (350 mV @ 10mA cm^−2^) [34], Ni_5_Mn-LDH (350 mV @ 10mA cm^−2^) [35], Co_5_Mn-LDH/MWCNT (300 mV @ 10mA cm^−2^) [35], NiMn LDHs nanosheets (320 mV @20mA cm^−2^) [36], Mo intercalated NiFe LDH (280 mV @ 10mA cm^−2^) [49], Ni_0.75_V_0.25_ LDH (315 mV @ 10mA cm^−2^) [50], Fe-doped NiV LDH (269 mV @ 10mA cm^−2^) [51], NiV LDH (319 mV @ 10mA cm^−2^) [52], NiCr LDH (319 mV @ 10mA cm^−2^) [53], and NiCo LDH (293 mV @ 10mA cm^−2^) [54]. Even at high current density, the Ni_0.65_Mn_0.35_ maintains improved catalytic performance to those of the other catalysts. Thus, the enhanced OER activity could be due to the synergistic effect of electronic structural modification and the porous-membrane-like structure [55,56,57].

**Figure 5:**
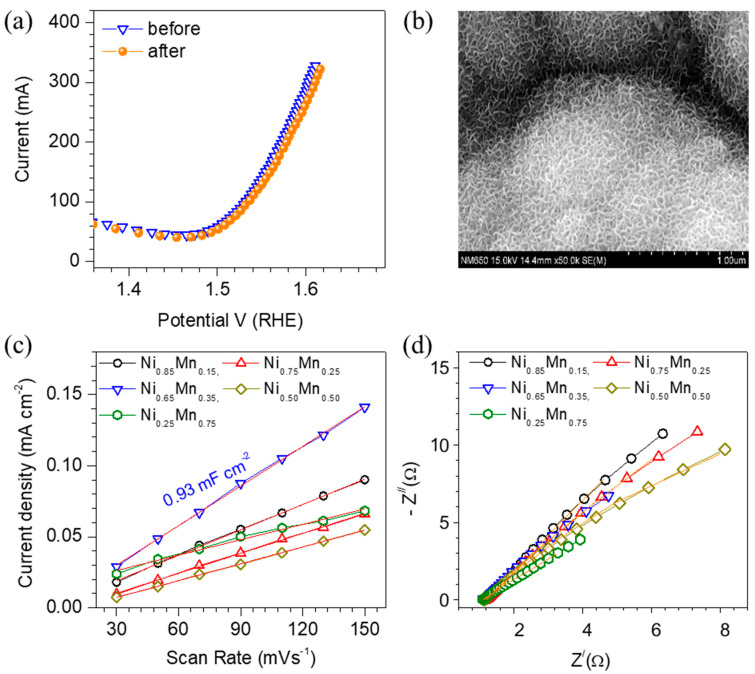
(**a**) The identical *iR*—corrected LSV curves of the best-performing Ni_0.65_Mn_0.35_; thin film electrode before and after the 20 h stability test confirms its excellent electrochemical stability. (**b**) No change in the SEM morphology after the stability test. (**c**) The slope of the capacitive current (Δj) measured at a non−Faradaic voltage versus the scan rate for the estimation of the double-layer capacitance and ECSA. (**d**) Nyquist plots for the Ni_1−x_Mn_x_ LDH thin film electrodes recorded at a 0-bias voltage using the equivalent circuit diagram used to fit the curves.

The Tafel slope is another important parameter to obtain more insight into the OER activity of the Ni_1−x_Mn_x_ LDH catalyst electrodes, which is evaluated from the LSV curves. Figure 4d displays a graph of overpotential (V) vs. log *j* (mA cm^−2^). The Tafel slopes are found to be 149, 138, 130, 134 and 142 mV dec^−1^ for the samples Ni_0.85_Mn_0.15_, Ni_0.75_Mn_0.25_, Ni_0.65_Mn_0.35_, Ni0_.50_Mn_0.50_ and Ni_0.25_Mn_0.75_, respectively. The lowest Tafel slope observed by the sample Ni_0.65_Mn_0.35_ agrees with the LSV results, suggesting that the sample has faster reaction kinetics, leading to a superior OER performance. Thus, the variation in the Tafel slope of the Ni_1-x_Mn_x_ LDH thin film catalyst electrodes indicates the alteration of the electronic structure and electronic conductivity. Further electrochemical stability in the alkaline electrolyte is a crucial parameter to develop high-performance catalysts for large-scale production. It is measured at current densities of 100 mA cm^−2^ for 20 h via the chronoamperometry (CA) technique. Figure 4e shows the CA curves (without *iR*-correction) for the different NiMn-LDH thin film electrodes. The Ni_0.65_Mn_0.35_ catalyst preserves the steadiest and lowest overpotential compared with the other catalysts for over 20 h of operation. Thus, the superior performance of the Ni_0.65_Mn_0.35_ could be associated with enhanced reaction kinetics and a large number of active sites. The negligible change in LSV (Figure 5a) behavior and morphology (Figure 5b) after the OER stability test indicates the excellent stability of the catalyst electrodes. Figure S6 depicts the SEM images of the best-performing Ni_0.65_Mn_0.35_ catalyst before and after the stability test. It is seen that the cracks become wider with the operation time; however, these cracks do not show any effect on the electrochemical performance of the catalyst, as shown in Figure 4e and Figure 5a, thus suggesting the outstanding electrochemical stability of the electrode for a long cycle life.

During the OER processes, catalysts undergo surface reconstruction; therefore, the surface characterization provides information about the active phases in the catalyst. To obtain details on the active sites in the NiMn LDH catalyst, we performed ex situ XPS measurements of the best-performing Ni_0.65_Mn_0.35_ after the OER test. Figure S7a–d show the XPS spectra of the Ni_0.65_Mn_0.35_ after 20 h of stability measurements in the alkaline electrolyte. Survey spectra shown in Figure S7a, exhibiting peaks of Ni, Mn, O and C in the sample, revealed no dissolution of any elements during OER. Noticeable changes in the core level spectra of Ni2p, Mn2p and O1s are observed. The intensity of the peak Ni2p_3/2_ in Figure S7b located at 854.8 eV increased considerably, which is associated with the existence of the Ni^2+^, along with the metal–hydroxide (M-OH, where M = Ni, Mn) peak at 530.62 eV in O1s in the spectra shown in Figure S7d. Moreover, a similar effect is observed for the peak intensity of the Mn^3+^ oxidation states (which is active for water oxidation) through the peak located at 641.5 eV in Mn 2p spectra (Figure S7c), suggesting the surface reconstruction of the catalyst electrode. Thus, the above analysis suggests that the catalyst transformed its surface into an active layer of nickel oxyhydroxide/hydroxide, which is one of the most catalytically active and stable phases.

The OER activities of the Ni_1−x_Mn_x_ LDH electrodes are discussed in terms of the electrochemical surface area (ECSA) and electrochemical impedance spectroscopy (EIS). It is mainly accredited to the following factors: the (1) charge transfer resistance, (2) ECSA, (3) ionic diffusion, (4) film thickness, (5) ionic conductivity, and (6) concentration of the electrolyte. However, in this study, the ionic diffusion, film thickness, ionic conductivity, and the concentration of the electrolyte are presumed to be constant for all of the samples. Therefore, it is important to estimate the ECSA and EIS, which are directly related to the OER activity of the NiMn LDH catalysts. To estimate the ECSA of the NiMn LDHs, a cyclic voltammetry (CV) technique is used. Figure S8 (Supplementary Information) shows the CV curves recorded in the non-Faradaic voltage region in the 1 M KOH electrolyte at 30, 50, 70, 90, 110, 130 and 150 mVs^−1^. The ECSA of the samples can be estimated using the following equation. The slope of the graph scan rate (mVs^−1^) versus the current density (mAcm^−2^) obtained from CVs provides a double-layer capacitance (C_dl_) [58].

ECSA = C_dl_/C_s_ (1)

*Cs* is referred to as the specific capacitance (0.040 mF cm^2^) for the KOH electrolyte. Therefore, the plot of the scan rate (mVs^−1^) versus the current density (mAcm^−2^) is plotted in which the current density is considered at a fixed potential of 0.1 V. The estimated C_dl_ and ECSA values of all of the samples are presented in Table S2 (Supplementary Information). As expected, the sample Ni_0.65_Mn_0.35_ exhibited the highest ECSA and C_dl_ values of 23.25 cm^2^ and 0.93 mF cm^−2^, respectively, suggesting the highest number of active sites on the surface of the catalyst electrode. Thus, the higher ECSA is indicative of the enhanced OER activity of the Ni_0.65_Mn_0.35_, which agrees with the LSV results. Moreover, to know the intrinsic OER activity, the LSV curves are normalized by dividing the current density axis with ECSA, as shown in Figure S9 (Supplementary Information). The ECSA normalized LSV curves suggest that the best-performing sample Ni_0.65_Mn_0.35_ has poorer OER activity than Ni_0.75_Mn_0.25_ and Ni_0.50_Mn_0.50_. This indicates that the enhanced OER activity in Ni_0.65_Mn_0.35_ is mainly contributed by the ECSA of the sample rather than other factors like the electrical conductivity.

Further, the electrical conductivity of the Ni_1-x_Mn_x_ LDH catalyst electrodes is investigated using EIS analysis by recording Nyquist plots, which are shown in Figure 5d. The significance of the semicircle and straight line is indicated by the charge-transfer resistance (*R*2) and Warburg impedance (*W*), respectively. To estimate these parameters along with the constant phase element (*CPE*) and solution resistance (*R*1), an equivalent circuit diagram, as shown in Figure S10 (Supplementary Information), is used to fit the Nyquist plots. The parameters obtained after fitting the Nyquist plots of all the samples are provided in Table S3 (Supplementary Information). It has been observed that the best-performing Ni_0.65_Mn_0.35_ sample has higher charge transfer resistance (19.55 Ω) than that of the Ni_0.75_Mn_0.25_ (11.45 Ω) and Ni_0.50_Mn_0.50_ (16.24 Ω), suggesting that the electronic conductivity is not the major factor to enhance the OER activity. Therefore, the tradeoff between ECSA and electrical conductivity of the Ni_0.65_Mn_0.35_ sample was likely the main reason for the enhanced OER activity.

## 4. Conclusions

In this work, we demonstrated the fabrication of the Ni_1−x_Mn_x_ LDH thin film catalyst electrodes onto a porous membrane, Ni foam, via a simple and cost-effective electrodeposition technique for effective utilization in the OER process. The relative composition between Ni and Mn and the porous-membrane-like structure played an important role in altering the morphology and electronic conductivity, thereby enhancing the OER performance. The optimized electrode with the compositional ratio of Ni_0.65_Mn_0.35_ with easy accessibility to the electrolyte exhibited excellent OER activity with the lowest overpotential of 253 mV at a current density of 50 mAcm^−2^. The low Tafel slope of 130 mV dec^−1^ shows faster and more favorable reaction kinetics of the catalyst. More interestingly, the sample is highly stable without any change in its shape and size after long-cycle life operation in an alkaline medium. The major contributor to the OER activity is ECSA, which gives an idea to design the other efficient catalyst for water electrolysis. Therefore, NiMn LDH has significant potential as an OER electrode in water electrolysis for hydrogen generation.

## Data Availability

Data Availability Statements are available in this article.

## Supplementary Materials

The following supporting information can be downloaded at: https://www.mdpi.com/article/10.3390/membranes13090748/s1, Figure S1. Chronoamperometric curves for the electrodeposition of the Ni_1-x_Mn_x_ LDH (where x = 0.15, 0.25, 0.35, 0.50 and 0.75) thin film electrodes. Figure S2. Typical XRD pattern of the Ni_0.65_Mn_0.35_ thin film electrode, with clear diffraction peak at 11.20°, 23.04°, and 34.46° suggesting formation of the NiMn LDH (JCPDS No. 38-0715) structure. Figure S3. low magnification SEM images of the Ni_1-x_Mn_x_ LDH thin film electrodes. (a) Ni_0.85_Mn_0.15,_ (b) Ni_0.75_Mn_0.25,_ (c) Ni_0.65_Mn_0.35_, (d) Ni_0.50_Mn_0.50_ and (e) Ni _0.25_Mn _0.75._ All the samples showed the existence of the cracks in the sample. Figure S4. Energy dispersive X-ray analysis (EDX) of the NiMn LDH thin film catalyst to detect the elemental composition. It suggests the presence of the Ni, Mn, and O in the sample. Figure S5. Enlarged view of the of the *iR*-corrected LSV curves of Ni_1-x_Mn_x_ LDH thin film electrodes. Figure S6. SEM images of the best performing Ni_0.65_Mn_0.35_ catalyst before and after stability test. Figure S7. X-ray photoelectron spectroscopy data of the best performing Ni_0.65_Mn_0.35_, thin film electrode after long term OER test of 20 hours. (a) survey spectra indicating presence of Ni, Mn, C and O in the catalysts, deconvoluted spectra of the (b) Ni 2p, (c) Mn 2p, (d) O 1s. Figure S8. CV curves recorded in the non-Faradaic voltage region in 1 M KOH electrolyte at different scan rates of 30, 50, 70, 90, 110, 130 and 150 mV s^−1^. (a) Ni_0.85_Mn_0.15,_ (b) Ni_0.75_Mn_0.25,_ (c) Ni_0.65_Mn_0.35_, (d) Ni_0.50_Mn_0.50_ and (e) Ni _0.25_Mn _0.75_. Figure S9. *iR*-corrected LSV curves of the Ni_1-x_Mn_x_ LDH thin film electrodes normalized with the ECSA values to know its intrinsic catalytic activity. Figure S10. Equivalent circuit diagram used to fit the Nyquist plots to estimate solution resistance (*R1*), charge transfer resistance (*R2*), Warburg impedance (*Wo*) and constant phase element (*CPE*). Table S1. Estimated OER parameters of the Ni_1-x_Mn_x_ LDH thin film electrodes in terms of the overpotential at different current densities and Tafel slopes. Table S2. Double layer capacitance and ECSA of the Ni_1-x_Mn_x_ LDH thin film electrodes estimated from the plot of the scan rate (mVs^-1^) versus current density (mAcm^−2^). Table S3. EIS parameters of the Ni_1-x_Mn_x_ LDH thin film electrode estimated after fitting of the Nyquist plots.
