# Supplementary material for: Nanoflake NiMn Layered Double Hydroxide Coated on Porous Membrane-like Ni-Foam for Sustainable and Efficient Electrocatalytic Oxygen Evolution
Source: Membranes (Basel). 2023 Aug 22;13(9):748. doi: 10.3390/membranes13090748 (PMC10535034; doi:10.3390/membranes13090748)
Supplement: Supplementary file 1 [file membranes-13-00748-s001.zip › membranes-2572827-supplementary.pdf]

membranes

Supplementary Materials

Nanoflake NiMn Layered Double Hydroxide Coated on Porous Membrane-like Ni-Foam for Sustainable and Efficient Electrocatalytic Oxygen Evolution

Verjesh Kumar Magotra [1], Arjun Magotra [2], Sawanta S. Mali [3], H. C. Jeon [1], T. W. Kang [1], Amol S. Salunke [4], Chang Kook Hong [3], Nabeen K. Shrestha [4], Hyunsik Im [4] and Akbar I. Inamdar [4,*]

[1] Quantum-Functional Semiconductor Research Center, Dongguk University, Seoul, Republic of Korea
[2] Department of Computer Science and Engineering, Faculty of Engineering and Technology, Jain (Deemed-to-be University), Bengaluru 562112, India
[3] Polymer Energy Materials Laboratory, School of Applied Chemical Engineering, Chonnam National University, Gwangju 500-757, Republic of Korea
[4] Division of Physics and Semiconductor Science, Dongguk University, Seoul 04620, Republic of Korea
* Correspondence: akbarphysics@dongguk.edu

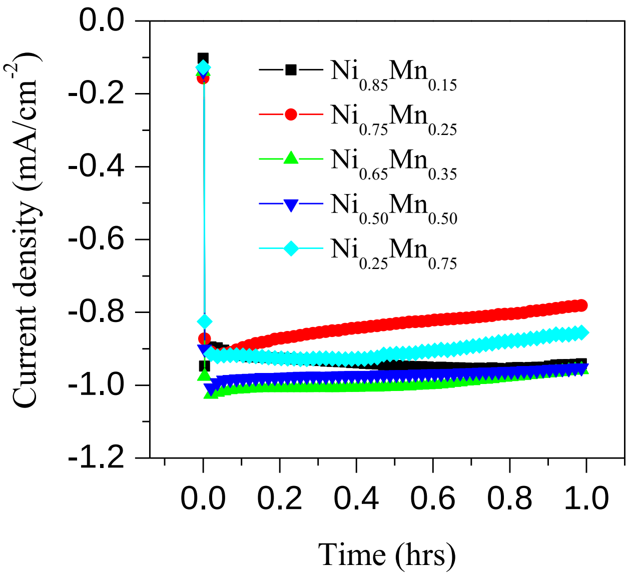

Figure S1. Chronoamperometric curves for the electrodeposition of the $Ni_{1-x}Mn_x$ LDH (where x = 0.15, 0.25, 0.35, 0.50 and 0.75) thin film electrodes.

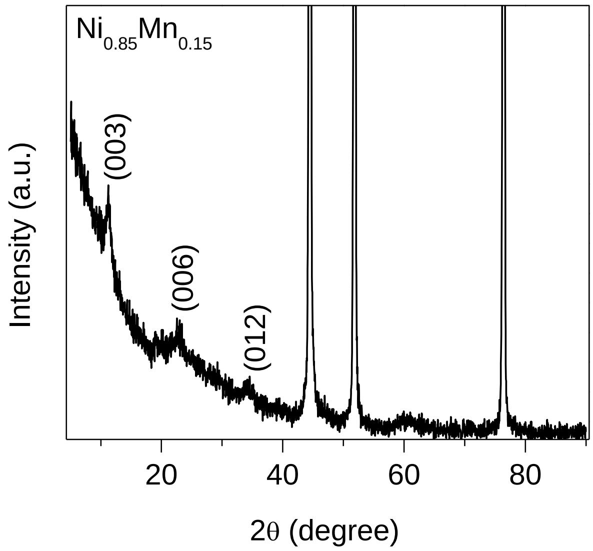

Figure S2. Typical XRD pattern of the $Ni_{0.65}Mn_{0.35}$ thin film electrode, with clear diffraction peak at 11.20°, 23.04°, and 34.46° suggesting formation of the NiMn LDH (JCPDS No. 38-0715) structure.

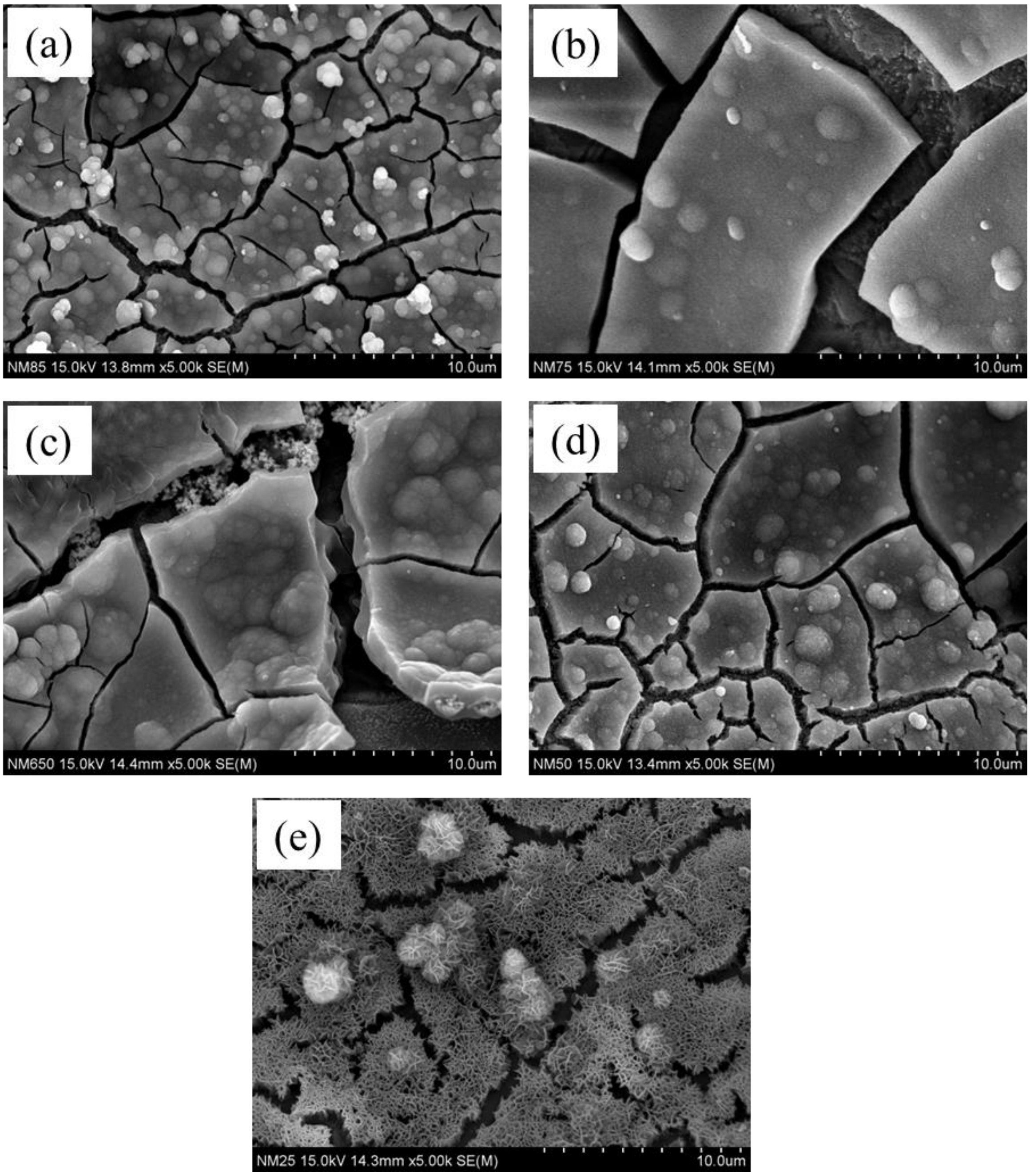

Figure S3. low magnification SEM images of the $Ni_{1-x}Mn_x$ LDH thin film electrodes. (**a**) $Ni_{0.85}Mn_{0.15,}$ (**b**) $Ni_{0.75}Mn_{0.25,}$ (**c**) $Ni_{0.65}Mn_{0.35}$, (**d**) $Ni_{0.50}Mn_{0.50}$ and (**e**) Ni $_{0.25}$Mn $_{0.75.}$ All the samples showed the existence of the cracks in the sample.

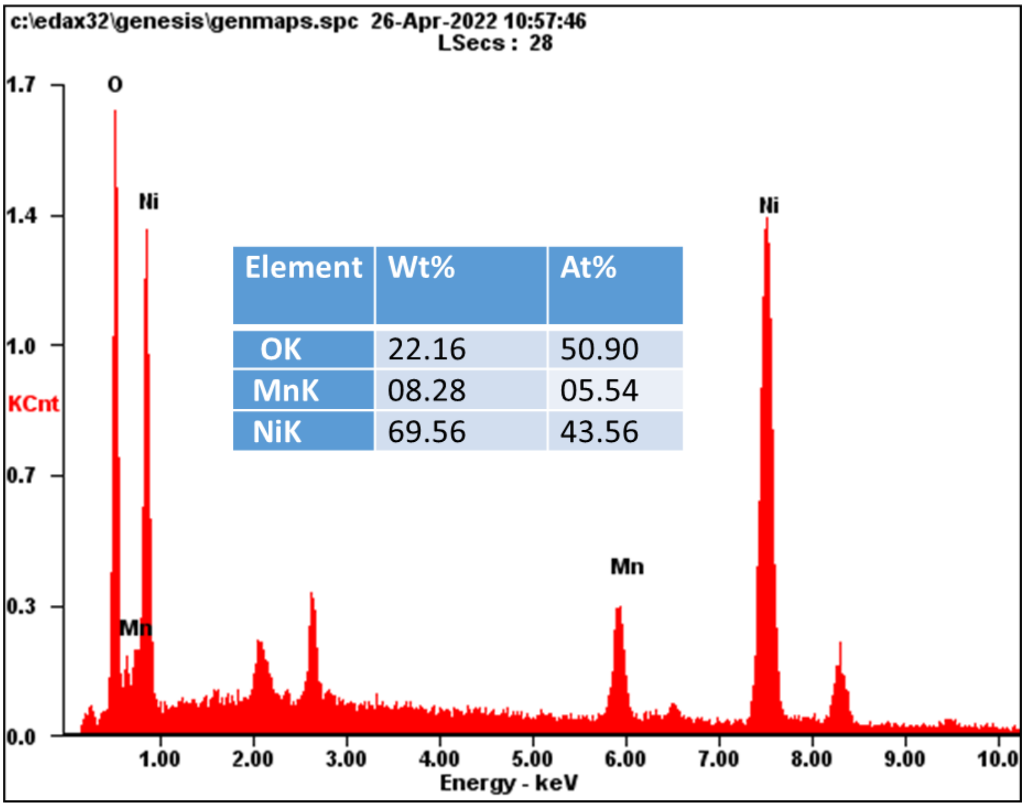

Figure S4. Energy dispersive X-ray analysis (EDX) of the NiMn LDH thin film catalyst to detect the elemental composition. It suggests the presence of the Ni, Mn, and O in the sample.

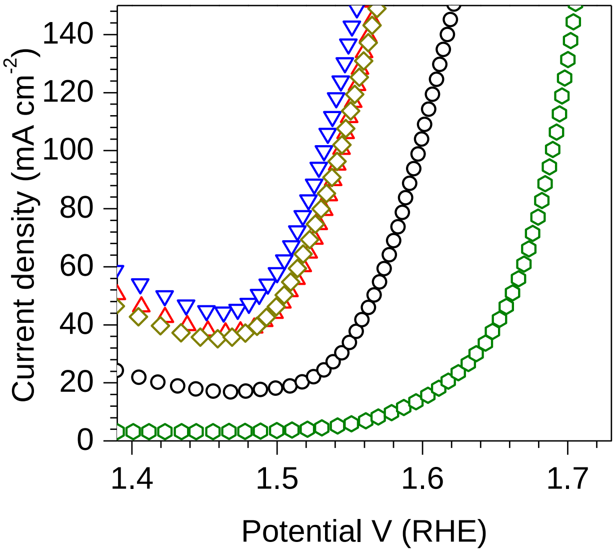

Figure S5. Enlarged view of the of the *iR*-corrected LSV curves of $Ni_{1-x}Mn_x$ LDH thin film electrodes.

Table S1. Estimated OER parameters of the $Ni_{1-x}Mn_x$ LDH thin film electrodes in terms of the overpotential at different current densities and Tafel slopes.

Catalysts	Overpotential (mV)			Tafel Slope
	50 mA cm^{-2}	100 mA cm^{-2}	200 mA cm^{-2}	mV dec^{-1}
**$Ni_{0.85}Mn_{0.15}$**	336	367	410	149
**$Ni_{0.75}Mn_{0.25}$**	277	315	350	138
**$Ni_{0.65}Mn_{0.35}$**	**253**	**299**	**342**	**130**
**$Ni_{0.50}Mn_{0.50}$**	268	310	359	134
**$Ni_{0.25}Mn_{0.75}$**	429	460	484	142

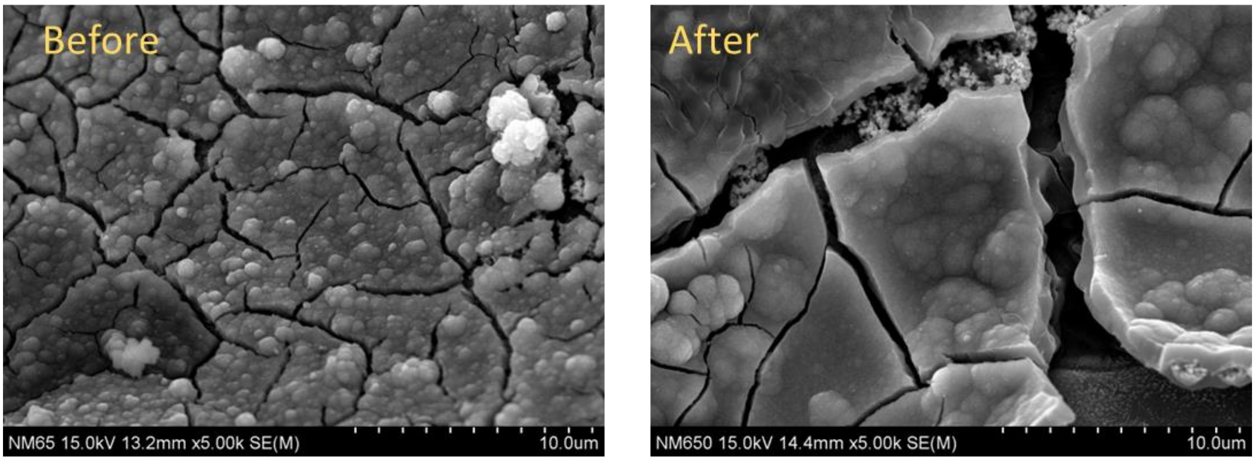

Figure S6. SEM images of the best performing $Ni_{0.65}Mn_{0.35}$ catalyst before and after stability test.

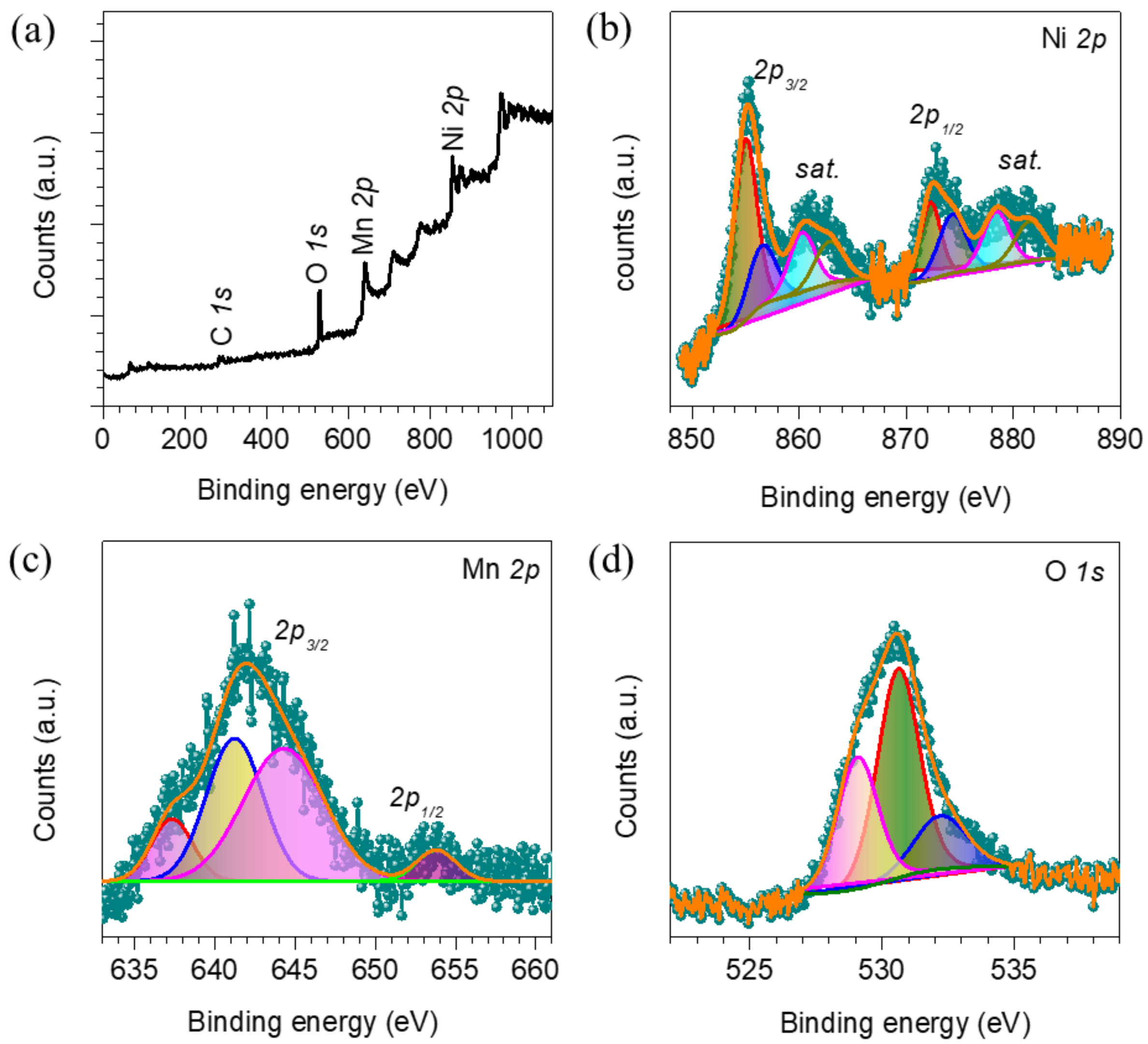

Figure S7. X-ray photoelectron spectroscopy data of the best performing $Ni_{0.65}Mn_{0.35}$, thin film electrode after long term OER test of 20 hours. **(a)** survey spectra indicating presence of Ni, Mn, C and O in the catalysts, deconvoluted spectra of the **(b)** Ni 2p, **(c)** Mn 2p, **(d)** O 1s.

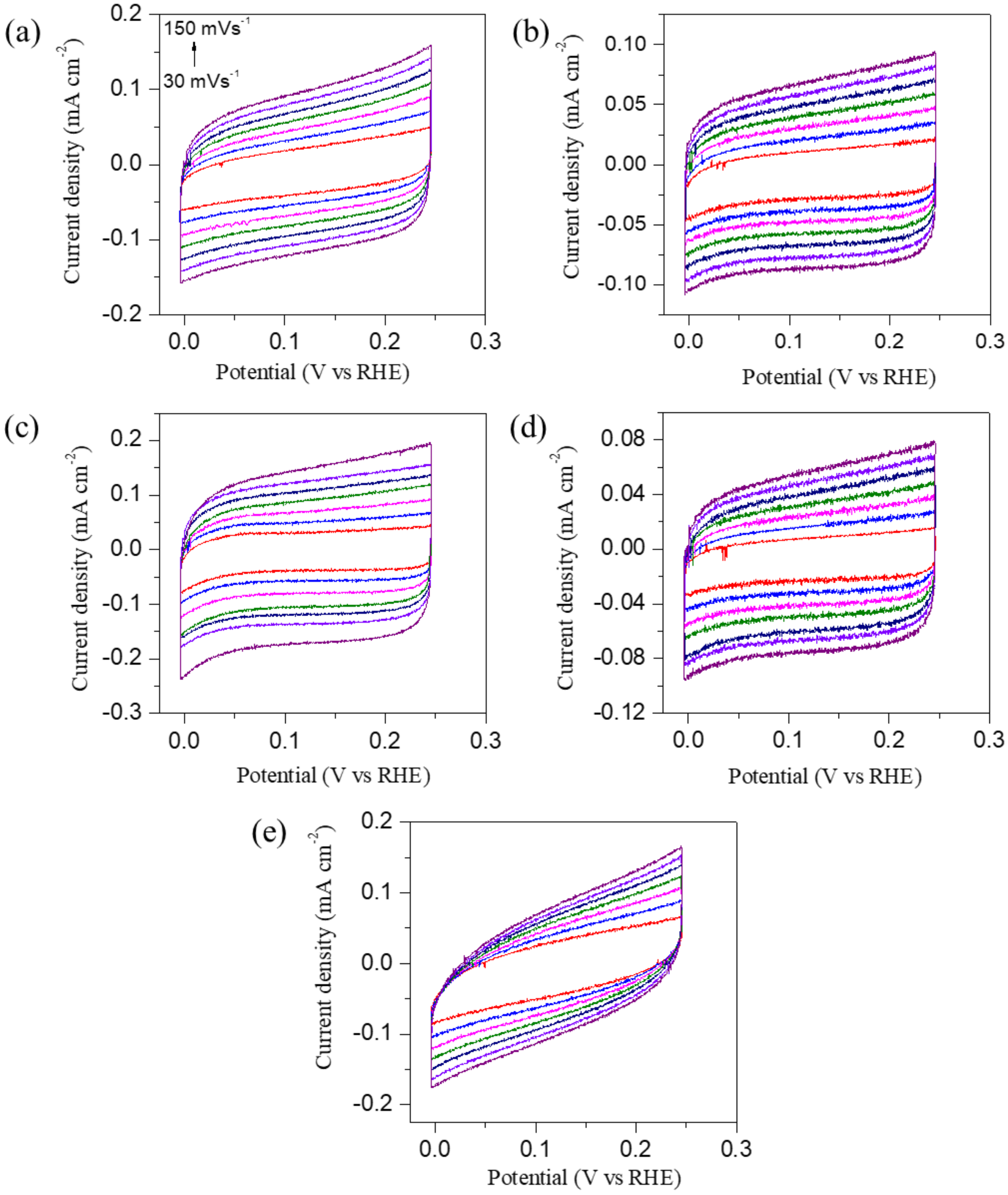

Figure S8. CV curves recorded in the non-Faradaic voltage region in 1 M KOH electrolyte at different scan rates of 30, 50, 70, 90, 110, 130 and 150 mV s^{-1}. **(a)** $Ni_{0.85}Mn_{0.15}$, **(b)** $Ni_{0.75}Mn_{0.25}$, **(c)** $Ni_{0.65}Mn_{0.35}$, **(d)** $Ni_{0.50}Mn_{0.50}$ and **(e)** Ni $_{0.25}$Mn $_{0.75}$.

Table S2. Double layer capacitance and ECSA of the $Ni_{1-x}Mn_x$ LDH thin film electrodes estimated from the plot of the scan rate (mVs^{-1}) versus current density ($mAcm^{-2}$).

Sample	Double-Layer Capacitances ($mF\ cm^{-2}$)	ECSA ($cm^{2)}$
**$Ni_{0.85}Mn_{0.15}$**	0.60	15.00
**$Ni_{0.75}Mn_{0.25}$**	0.49	12.25
**$Ni_{0.65}Mn_{0.35}$**	**0.93**	**23.25**
**$Ni_{0.50}Mn_{0.50}$**	0.39	9.75
**$Ni_{0.25}Mn_{0.75}$**	0.36	9.00

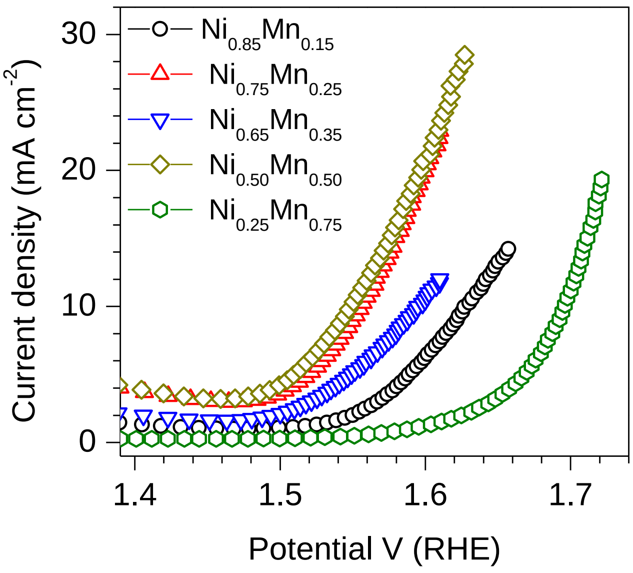

Figure S9. *iR*-corrected LSV curves of the $Ni_{1-x}Mn_x$ LDH thin film electrodes normalized with the ECSA values to know its intrinsic catalytic activity.

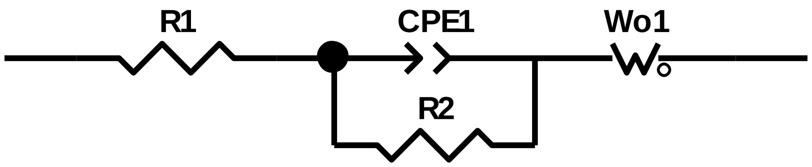

Figure S10. Equivalent circuit diagram used to fit the Nyquist plots to estimate solution resistance (*R1*), charge transfer resistance (*R2*), Warburg impedance (*Wo*) and constant phase element (*CPE*).

Table S3. EIS parameters of the $Ni_{1-x}Mn_x$ LDH thin film electrode estimated after fitting of the Nyquist plots.

Sample	*R1*(Ω)	*R2* Ω	*CPE*
$Ni_{0.85}Mn_{0.15}$	1.19	83.56	0.85
$Ni_{0.75}Mn_{0.25}$	1.13	11.45	0.79
$Ni_{0.65}Mn_{0.35}$	1.11	19.55	0.72
$Ni_{0.50}Mn_{0.50}$	1.12	16.24	0.85
$Ni_{0.25}Mn_{0.75}$	1.08	22.68	0.63
